# Movement decline across lifespan of *Caenorhabditis elegans* mutants in the insulin/insulin‐like signaling pathway

**DOI:** 10.1111/acel.12704

**Published:** 2017-12-07

**Authors:** Breanne L. Newell Stamper, James R. Cypser, Katerina Kechris, David Alan Kitzenberg, Patricia M. Tedesco, Thomas E. Johnson

**Affiliations:** ^1^ Institute for Behavioral Genetics University of Colorado Boulder Boulder CO USA; ^2^ Department of Integrative Physiology University of Colorado Boulder Boulder CO USA; ^3^ Department of Biostatistics and Informatics University of Colorado Denver Denver CO USA

**Keywords:** aging, *C. elegans*, demography, healthspan, insulin signaling, life‐history, lifespan, longevity, movement, mutants

## Abstract

Research in aging biology has identified several pathways that are molecularly conserved across species that extend lifespan when mutated. The insulin/insulin‐like signaling (IIS) pathway is one of the most widely studied of these. It has been assumed that extending lifespan also extends healthspan (the period of life with minimal functional loss). However, data supporting this assumption conflict and recent evidence suggest that life extension may, in and of itself, extend the frail period. In this study, we use *Caenorhabditis elegans* to further probe the link between lifespan and healthspan. Using movement decline as a measure of health, we assessed healthspan across the entire lifespan in nine IIS pathway mutants. In one series of experiments, we studied healthspan in mass cultures, and in another series, we studied individuals longitudinally. We found that long‐lived mutants display prolonged mid‐life movement and do not prolong the frailty period. Lastly, we observed that early‐adulthood movement was not predictive of late‐life movement or survival, within identical phenotypes. Overall, these observations show that extending lifespan does not prolong the period of frailty. Both genotype and a stochastic component modulate aging, and movement late in life is more variable than early‐life movement.

## 1 INTRODUCTION

Aging, the progressive loss of physiological function that occurs with time (Finch, 1990) is a huge burden on society. Aging is one of the largest risk factors for chronic diseases including, but not limited to, cancer, arthritis, diabetes, and Alzheimer's (López‐Otín, Blasco, Partridge, Serrano, & Kroemer, 2013). Consequently, the majority of healthcare costs arise during the last few years of life. The loss of function and increased vulnerability to disease and death leads to reduced independence and quality of life. The proportion of the population reaching older ages is increasing globally. If unaddressed, the burden of healthcare costs could lead to economic disaster (Olshansky, Goldman, Zheng, & Rowe, 2009). Hence, there is a strong motivation to curb the age‐associated loss of function, not only to avoid economic disaster, but also to improve quality of life for aging individuals and their families (Goldman et al., 2013).

In the last few decades, aging research has largely focused on modulation of lifespan and has been successful in extending lifespan in multiple model systems, including yeast, worms, and flies (Kirkland & Peterson, 2009). Many longevity genes, with natural functions that inhibit life extension, have been identified. In fact, the first metazoan longevity gene, *age‐1,* was identified in *C. elegans*. The *age‐1/*PI3K ortholog is involved in the insulin/insulin‐like signaling (IIS) pathway and *age‐1* mutants live 40–60% longer than wild‐type controls (Friedman & Johnson, 1988). Other mutations in the IIS pathway have been shown to increase lifespan. Notably, the *daf‐2*/IGF‐1 receptor mutant has a lifespan more than double that of wild‐type (Kenyon, Chang, Gensch, Rudner, & Tabtlang, 1993). The genes of the IIS pathway are molecularly highly conserved across species, including mammals (Murphy & Hu, 2013), making the nematode a valuable tool for aging research.

While the field has been successful in identifying genetic, behavioral, and pharmacological interventions that modulate lifespan, less attention has been directed at “healthspan.” Healthspan is a newly coined word meaning the adult period of unimpaired activity and function that precedes age‐related decline (Herndon et al., 2002). Do mutations that extend lifespan delay or slow aging, thereby delaying or slowing a broad spectrum of age‐related impairments? Many studies support the observations showing that longevity mutants delay loss of neuromuscular function, slow morphological decrepitude, and delay molecular changes (Collins, Huang, Hughes, & Kornfeld, 2007). Research from our laboratory and others demonstrates that organisms that are long‐lived are also resistant to multiple stressors (UV, oxidative stress, heavy‐metal toxicity, and heat stress), further supporting the positive relationship between longevity and robustness (Johnson et al., 2000).

Some aspects of the relationship between lifespan and healthspan have been identified, although results conflict and healthspan in older animals was often not assessed. Recently, several studies revisited the question “Do longevity mutants extend healthspan in *C. elegans*?”. Bansal, Zhu, Yen, and Tissenbaum (2015) studied multiple physiological parameters over the lifespan of several longevity mutants. They found that long‐lived mutants extend chronological healthspan but when normalized to lifespan, long‐lived mutants actually produce a longer period of frailty (Bansal et al., 2015). Hahm et al. (2015) studied maximum velocity of worm movement in *daf‐2(e1370)* mutants and reevaluated the data from Bansal et al. (2015). They found that some longevity mutants (e.g., *clk‐1)* extend frailty, but others (e.g., *daf‐2)* do not. Corroborating these findings, Podshivalova, Kerr, and Kenyon (2017) found that two *daf‐2* mutants have extended movement ability and that the *daf‐2(e1370)*, but not the *daf‐2(e1368)* allele, has reduced movement velocity. Taken together, the comodulation of lifespan and healthspan remains murky. Two conceptual models of healthspan seem to be emerging. The first we call “the rate of aging model” wherein the underlying expectation is that aging is a process still governed by unidentified events underlying both health and longevity, resulting in a positive correlation between healthspan and lifespan. The second we call “the medical model” which puts forth the notion that the processes that govern longevity can be uncoupled from the processes that govern healthspan, thus resulting in an extension of frailty.

The purpose of this study was to elucidate the relationship between lifespan and healthspan in *C. elegans*. We studied movement across lifespan of nine IIS pathway mutants. Movement was assessed in two types of samples: mass populations and individual worms. In both approaches, movement was assessed longitudinally from early adult until 100% of the population had died. The strength of this approach is that multiple long‐lived mutants can be studied simultaneously, providing a longitudinal assessment of movement decline.

## 2 RESULTS

### 2.1 Many, but not all, IIS mutants have extended lifespan

**Figure 1:**
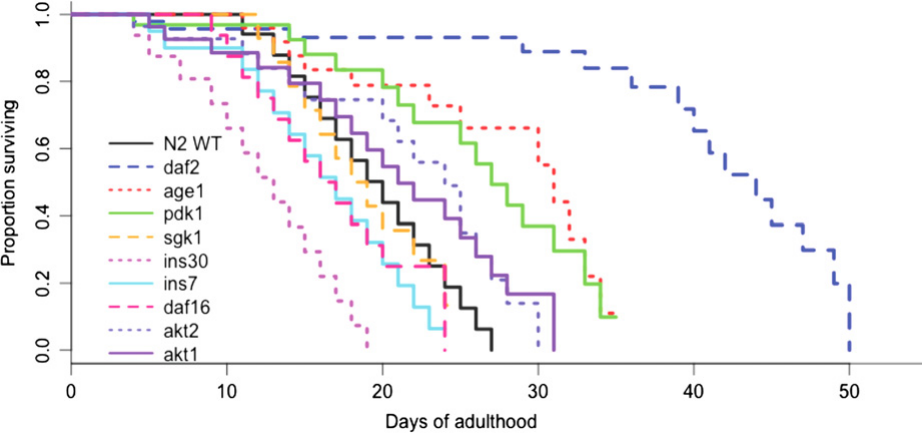
Survival curves for IIS mutants. Curves are the pooled survival data for a given mutant. See Table 1 for numerical details

To assess the effects of IIS mutations on lifespan, we carried out survival experiments on nine single‐gene IIS mutants: *age‐1(hx546), akt‐1(mg306), akt‐2(ok393), daf‐2(e1370), daf‐16(mu86), ins‐7(ok1573), ins‐30(ok2343), pdk‐1(sa680),* and *sgk‐1(ok538)*. All mutants had previously been reported to extend lifespan (Friedman & Johnson, 1988; Hertweck, Gobel, & Baumeister, 2004; Kenyon et al., 1993; McElwee, Bubb, & Thomas, 2003; Murphy, Lee, & Kenyon, 2007; Paradis, Ailion, Toker, Thomas, & Ruvkun, 1999; Tullet et al., 2008; Zhang et al., 2008). However, the *sgk‐1* mutant has been observed to have variable lifespan (Chen, Guo, Dumas, Ashrafi, & Hu, 2013; Mizunuma, Neumann‐Haefelin, Moroz, Li, & Blackwell, 2014; Xiao et al., 2013). We used the log‐rank test to analyze survival. Consistent with what is reported in the literature, the mutants *daf‐2, age‐1, pdk‐1, akt‐1,* and *akt‐2* have significantly extended lifespans (Figure 1, Table 1). *age‐1* had a mean lifespan of 26.5 days and a maximum lifespan of 35 days (*p *= .009). *daf‐2* had the greatest extension in lifespan with a mean lifespan of 29.5 days and a maximum lifespan of 50 days (compared to a 14.5 day mean and 27 day max. lifespan for wild‐type, *p *< .001). The *akt‐1* and *akt‐2* mutants had mean lifespans of 20.0 and 15.0 days, and a maximum lifespan of 31 days (*p *= .004 and 0.013, respectively, Table 1). The *pdk‐1* mutant had a robust extension of mean and maximum lifespan, 19.5 days mean lifespan, and 35 days maximum lifespan (*p *< .022, Table 1).

**Table 1:** Lifespan (LS) of IIS mutants

Strain	Med (days)	Mean (days)	SE (days)	Max LS Observed Max LS from replicates (days)	*p*‐value	No. uncensored	No. censored	No. cohorts	*N*
*age‐1(hx546)*	31.0	26.5	0.14	35 (35,25,31,32)	.0093	30	22	3	52
*akt‐1(mg306)*	21.0	20.0	0.11	31 (29,26,31,28)	.004	62	19	4	81
*akt‐2(ok393)*	24.0	15.0	0.12	31 (30,26,31)	.013	38	17	3	55
*daf‐16(mu86)*	16.5	13.5	0.13	24 (19,22,24,19,18,22)	.0004	115	5	6	120
*daf‐2(e1370)*	44.0	29.5	0.12	50 (50,45,44,44,50,45)	3.3 × 10^−13^	114	5	6	119
*ins‐30(ok2343)*	13.0	13.5	0.13	19 (17,19)	1.9 × 10^−13^	54	3	2	39
*ins‐7(ok1573)*	17.0	16.5	0.12	25 (25,20)	5.8 × 10^−6^	35	4	2	39
N2 (control)	20.0	14.5	0.13	27 (20,25,26,27,27,27,17,25,22,15)	–	157	31	10	188
*pdk‐1(sa680)*	27.0	19.5	0.12	35 (35,27,30,30)	.022	58	15	4	73
*sgk‐1(ok538)*	18.5	15.5	0.13	21 (20,17,25,19)	.0045	63	15	5	78

Days are days of adulthood. Maximum lifespan was the last day a worm was alive. The log‐rank analysis was used to determine whether lifespan differs between a mutant and the control strain (N2). Survival data from all experiments and all experimenters were pooled for the analysis. Median lifespan (Med.LS), mean lifespan (Mean LS), standard error (SE), and maximum lifespan (Max. LS) are listed. The maximum lifespans from each replicate are included.

### 2.2 Some IIS mutants are short‐lived

Contrary to what was reported in the literature, mutations in either of two insulin‐like peptides (*ins‐7* and *ins‐30*) upstream of *daf‐2* did not increase lifespan in our hands (Figure 1 and Table 1). *ins‐30* was significantly short‐lived (13.5 days mean lifespan, 19 days max. lifespan, *p *< .001). Similarly, the *ins‐7* mutant had a mean lifespan of 16.5 days and a maximum lifespan of 25 days (*p *< .001). Downstream of *daf‐2, age‐1,* and *pdk‐1*, the SGK‐1 protein complexes with AKT‐1/AKT‐2 proteins to phosphorylate DAF‐16. Disruption of this process would be expected to extend lifespan, and indeed RNAi for *sgk‐1* has been reported to extend lifespan (Hertweck et al., 2004). In other reports, the *sgk‐1* mutant is short‐lived (Chen et al., 2013; Xiao et al., 2013). Mizunuma et al. (2014) report that the lifespan of *sgk‐1* mutants is dependent on environmental conditions (food and temperature). In our hands, the *sgk‐1* mutants had a shortened lifespan (15.5 days mean lifespan, 25 days maximum lifespan, *p *= .005). The life‐extending effect of mutating the IIS pathway is dependent on the DAF‐16/FOXO transcription factor remaining outside the nucleus (Gottlieb & Ruvkun, 1994; Kenyon et al., 1993; Murakami & Johnson, 1996; Ogg et al., 1997; Vowels & Thomas, 1992). Reducing *daf‐16* expression shortens lifespan in worms (Larsen, Albert, & Riddle, 1995; Ogg et al., 1997). As previously reported, the *daf‐16(mu86)* mutant was short‐lived (mean lifespan 13.5 days, max. lifespan 24 days, *p *< .001).

### 2.3 Movement decline is progressive

Diminishing movement as a function of age has been documented in multiple studies (Bolanowski, Russell, & Jacobson, 1981; Glenn et al., 2004; Herndon et al., 2002; Huang, Xiong, & Kornfeld, 2004; Johnson, 1987). Here, we use a method similar to that of Hosono, Sato, Aizawa, and Mitsui (1980) and Herndon et al. (2002), which categorizes movement into one of three classes, A, B, and C. All animals begin adult life with Class A movement, a marker of a vigorous, youthful state with minimal functional decline. Class B movement begins during mid‐life and is characterized by a loss of spontaneous movement, while the ability to respond to touch is maintained, indicating a decline in the function of body wall muscles (Herndon et al., 2002). Class C is observed later in life and in close proximity to death. This class is a biologically frail state characterized by significant muscle function decline and a complete lack of body movement with some head and tail movement. It has been observed that long‐lived mutants display slowed‐aging phenotypes, including delayed onset of movement decline (Herndon et al., 2002).

In our experiments, we classified movement every 1–3 days, beginning on the second day of adulthood (day 5 of life for most strains). In this first series of studies, we followed populations of worms. For each observation, the number of worms in a given movement class (A, B, or C) was recorded. Movement differences between wild‐type (N2) and mutant strains were assessed by binomial logistic regression. The output of this analysis is the odds ratio of a mutant being in a movement class at a given time compared to N2. Additionally, contributions by covariates (age, experiment, experimenter) to variation in movement can be determined. As emphasized by Bansal et al. (2015), Hahm et al. (2015), and Churgin et al. (2017), when determining whether an intervention extends healthspan, it is important to account for lifespan differences across strains. Bansal et al. (2015) refer to this as the physiological versus chronological age or healthspan:gerospan ratio, and others refer to this as “healthspan scaling” (Churgin et al., 2017; Hahm et al., 2015). We accounted for such differences by including lifespan as a covariate in the regression analysis so that any remaining statistical differences in movement are attributable to genotype. We also tested for interactions between genotype and lifespan contributing to variation in movement class. A significant interaction indicates that the slope of the regression is different between a mutant and N2. In this context, it means the time a mutant spends in a movement class differs from N2 in an age‐dependent manner.

**Figure 2:**
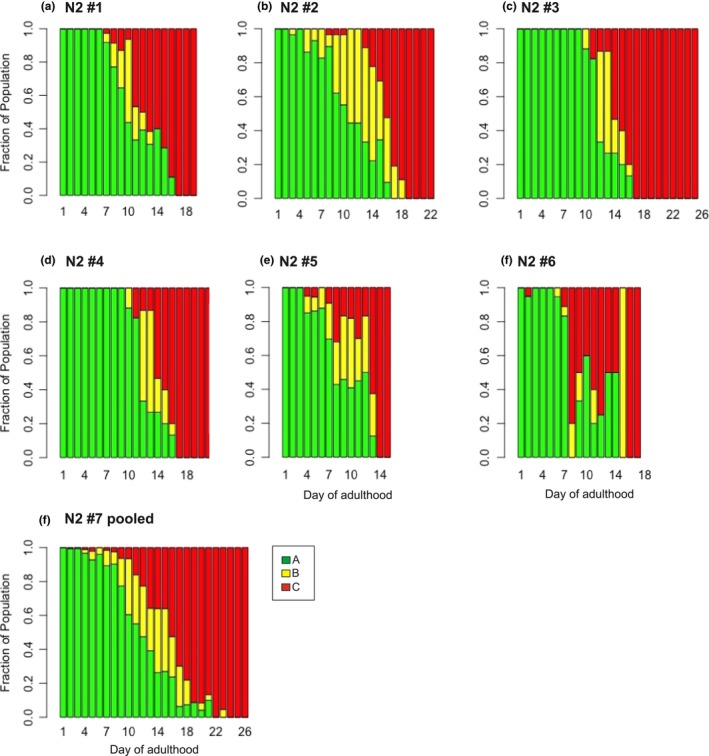
Movement Class as a function of age in the control N2 strain. Every 1–3 days of adulthood worms were scored for movement Class (A, B, or C). Green, yellow, and red bars represent the fraction of worms displaying Class A, B, or C movement, respectively, on a given day. The y‐axis shows fraction of each movement class, as a function of age. Panels a through f show data from individual experiments. Panel g shows the averaged data from panels a through e

**Figure 3:**
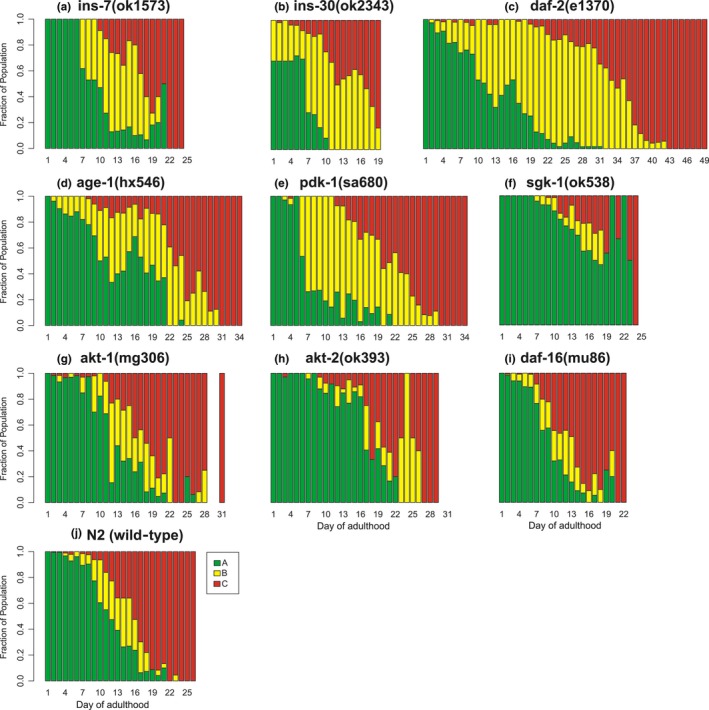
Movement Class as a function of age in IIS mutants. Data collected and analyzed as for Fig. 2. Data are pooled across experiments. The sample size and number of cohorts for each mutant are listed in Table 1. For statistical details, see Table S1. The mutants are ordered by their respective order in the IIS pathway

As is commonly reported, variation across experiments was observed. Data from all replicates were pooled for the analysis (Figure 2). Corroborating what has been reported in the literature, we observe that Class A (spontaneous) behavior declines significantly with age for all strains (*p *< .01, Figure 3 and Table S1) and that movement classes B and C significantly increase with age (*p *< .01; Figure 3 and Table S1).

### 2.4 Long‐lived mutants do not show significant changes in Class A “youthful” movement

Long‐lived mutant populations in this study (*daf‐2, age‐1, pdk‐1, akt‐1, akt‐2)* do not have prolonged Class A movement (Figure 3, Table S1). However, there is a significant interaction between genotype and age on Class A for *akt‐2* and *pdk‐1* (*p *< .01 for both, Table S1). The interaction reflects the larger fraction of *akt‐2* mutants with Class A movement between days 1–23 of adulthood as compared to N2. However, similar to N2, by day 23, all worms had lost Class A movement (Figure 3). The reverse was observed for *pdk‐1*. A smaller fraction of *pdk‐1* mutants maintained Class A movement for the first 23 days of adulthood, but that smaller fraction of *pdk‐1* mutants had prolonged Class A compared to N2. With the exception of *pdk‐1,* in our hands, life‐extending mutations neither preserve nor reduce early‐life, youthful movement.

### 2.5 Long‐lived mutants have large increases in Class B “middle‐aged movement” and decreases in “frail” movement (Class C)

The mutants with the greatest life extension (*age‐1, daf‐2,* and *pdk‐1*) display large increase in Class B movement (*p *< .05; Figure 3 and Table S1). For *age‐1, daf‐2*, and *pdk‐1,* the odds of being in Class B were 633%, 651%, and 432% higher than that for wild‐type (N2). The interactions between genotype and lifespan on Class B movement for these strains, as well as *akt‐1*, are significant (*p *< .05, Table S1). Thus, the effect of age on Class B movement differs in these long‐lived strains compared to wild‐type. Surprisingly, the long‐lived *akt‐2* reduces Class B movement (*p *< .002, Figure 3 and Table S1) regardless of age. These observations show that long‐lived mutants of the IIS pathway have extended periods of the intermediate movement behavior, Class B.

We expected to see a decrease in Class C movement for long‐lived mutants based on the findings of Herndon et al. (2002) and the rationale that longer‐lived mutants delay frailty/compress morbidity. Onset of Class C was the criteria we used to define frailty/morbidity. In long‐lived mutants, we did not observe an effect of genotype alone on Class C. There were significant interactions of genotype and age for *age‐1, daf‐2, pdk‐1,* and *akt‐1,* indicating Class C is reduced in these mutants depending on age (*p *< .05, Table S1). These data support the notion that mutations of the IIS pathway that extend lifespan are not associated with an increase in the odds of frailty, Class C (Table S1).

### 2.6 Short‐lived mutants trend toward a reduction in youthful movement and increased middle‐age and frail movement

Parallel to long‐lived mutants, short‐lived mutants (*ins‐7, ins‐30, daf‐16,* and *sgk‐1)* display a similar pattern of movement loss across lifespan. Short‐lived mutants tend to decrease Class A movement, and to increase both Class B and Class C. Almost all of the changes in movement observed in short‐lived mutants were nonsignificant (Table S1) with the exception of the *ins‐30* mutant. *ins‐30* mutants *do* significantly increase Class C. Even the well‐studied short‐lived *daf‐16* mutant lacked altered movement decline when movement was scaled to lifespan (variance in movement due to a shorter lifespan is accounted for). There were significant interactions between genotype and lifespan for *ins‐7, ins‐30,* and *daf‐16* on Class A (*p *< .01). These data demonstrate that most short‐lived mutants have unaltered healthspan when scaled to lifespan (Figure 3, Table S1). The exception to this was the *ins‐30* mutant*,* which was frailer than N2.

A notable exception to the pattern of movement for short‐lived mutants was the *sgk‐1* mutant. *sgk‐1* displayed extended Class A, and significantly less Class B and Class C movement than wild‐type (*p *< .05, Figure 3, Table S1). A significant interaction between genotype and age was observed for classes A and B movement (*p *< .01, Figure 3, Table S1). While short‐lived in these experiments, the lifespan of *sgk‐1* mutants is variable which may explain the extended healthspan of this mutant.

### 2.7 Longitudinal assessment of individual worms

**Figure 4:**
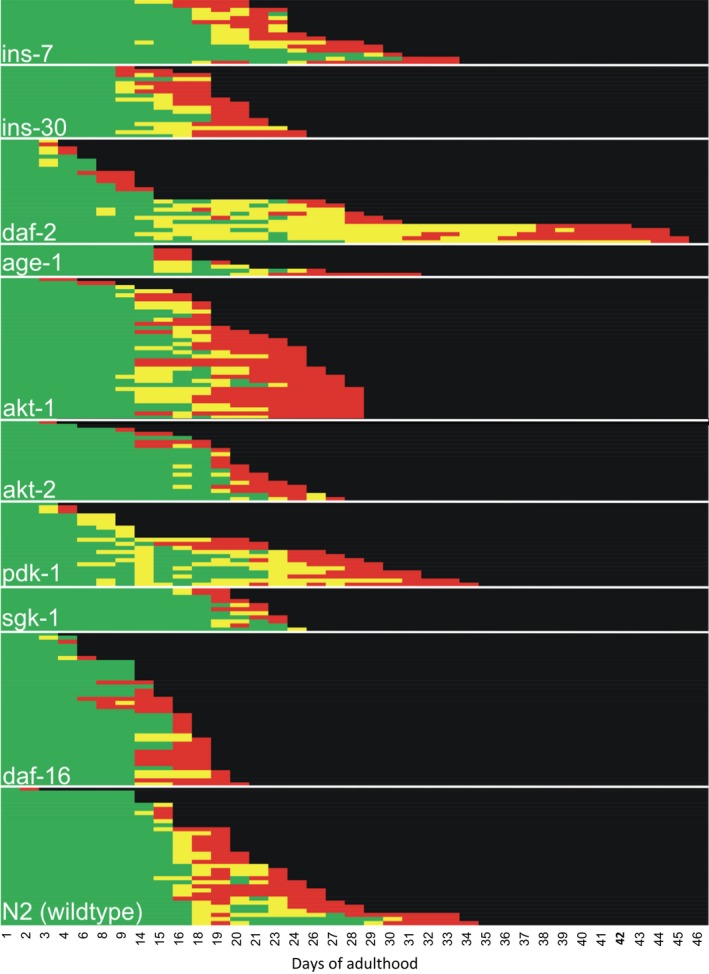
Movement classes for individual worms as a function of age. Each horizontal line is a worm's movement life history. Green, yellow, and red boxes represent Class A, B, or C movement, respectively. For statistical details, see Supplementary Table 2

Previous studies have shown that the age‐associated decline in movement not only varies across strains, but also within strains (Churgin et al., 2017; Herndon et al., 2002). The age of onset and the rate of movement decline vary widely among individual worms within a population (Churgin et al., 2017; Herndon et al., 2002). We visualized movement decline in individual worms across lifespan using event‐history plots (Figure 4; Carey, Liedo, Muller, Wang, & Vaupel, 1998). An event‐history plot portrays data at the individual level and allows visual comparisons of detailed life‐history patterns such as movement decay (Carey et al., 1998). Longitudinal assessment of individual worms is important because it reveals within‐strain variation in movement decline and allows for quantification of days spent in a movement class. As expected, we observed variation in movement decline within mutant populations (Figure 4).

### 2.8 Increasing lifespan did not increase the frailty in individual mutants

Longitudinal assessment of movement in individual worms allows us to ascertain the mean number of days an individual spent in a given movement class (Figures 3 and 4, Table S2), as opposed to a fraction of the population in a given movement class. A two‐tailed t‐test was used to assess differences in mean number of days spent in a class. For N2, the mean number of days spent in Class A was 9.3 days (SD = 3.4 days), 1.8 days (SD = 1.5 days) in Class B, and 2.1 days (SD = 1.8 days, *n *= 59) in Class C (Table S2). Both *daf‐2* and *pdk‐1* had large increases in the mean number of days spent in Class B. *daf‐2* increases Class B by 230% (6.0 days, SD = 8.3, *p *< .01, *n *= 25) and *pdk‐1* by 278% (6.8 days, SD = 5.7 days, *p *< .001, *n *= 21; Table S2). *age‐1* spent 55% more days in Class A than N2 (14.4 days, SD = 2.1 days, *n *= 9, *p *< .01; Table S2) but did not alter Class B or C. The *akt‐1* mutants spend more time in Class A (10.9 days, SD = 4.1, *p *= .02, *n *= 55) and, notably, Class C (4.0 days, SD = 3.5 days, *p *< .01). This was the only long‐lived mutant displaying extended frailty. An *akt‐2* mutation decreases time in Class B (1.1 days, SD = 1.2 days, *p *< .01, *n *= 32; Table S2). With the exception of *akt‐1*, our longitudinal assessments of movement in individual long‐lived mutants support an increase in healthspan by increasing the number of days in Class A or Class B.

For the mutants that were short‐lived, we observed altered duration of movement classes in the individual worm assessments. *daf‐16* spends less time in Class B (mean 1.0 day, SD = 1.6 days *p *< .01, *n *= 38) and does not extend the frailty period (2.1 days in Class C for *daf‐16* and N2). The *ins‐7* mutant (which was short‐lived in our hands) surprisingly increases Class A by 62% (15.1 days, SD = 4.3 days, *p *< .001, *n *= 16; Table S2). Both *ins‐7* and *ins‐30* mutants increase frailty (3.9 and 3.4 days, SD = 3.1 and 2.1 days, *p *< .01). *sgk‐1* had a 57% increase in days spent in Class A (mean 16.6 days, SD = 2.5 days, *p *< .001, *n *= 11; Table S2). Overall, shortening lifespan increases frailty in *ins‐7* and *ins‐30* mutants, but otherwise does not extend frailty for short‐lived mutants *sgk‐1* or *daf‐16*.

**Figure 5:**
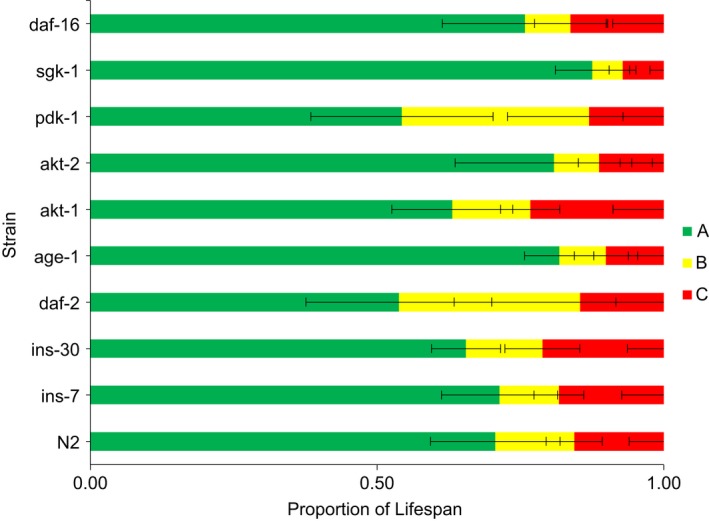
Proportion of lifespan in a movement class. Data show the proportion of life in a movement class (mean lifespan/mean days of movement class). For statistical support, see Table 2

To compare time spent in a movement class while accounting for differences in lifespan, we normalized mean number of days in a class to mean lifespan for that mutant (Table 2, Figure 5). This allowed us to visualize the healthspan:gerospan ratio utilized by Bansal et al. (2015); however, they normalized to maximal lifespan. No statistical comparison was made on this ratio (Figure 5). Wild‐type (N2) spent 62% of life in Class A, 12% of life in Class B, and 14% of life in Class C (Figure 5; Table 2). For most mutants, altered lifespan does not proportionally alter healthspan or frailty. Life extension is associated with Class B extension (Figure 5, Table 2).

**Table 2:** Proportion of life spent with a movement class. Using the movement data collected on individual worms, we divided mean number of days spent in a movement class by the mean lifespan for that mutant

Mutant	Class A	SD (days)	*p*‐value	Class B	SD (days)	*p*‐value	Class C	SD (days)	*p*‐value
*age‐1*	0.82	2.1	**.00**	0.08	1.9	.50	0.10	2.2	.68
*akt‐1*	0.56	4.1	**.02**	0.12	2.0	.10	0.21	3.5	**.00**
*akt‐2*	0.68	5.5	.07	0.07	1.2	**.00**	0.09	1.8	.12
*daf‐16*	0.76	3.6	.70	0.08	1.6	**.01**	0.16	2.2	1.0
*daf‐2*	0.54	6.2	.38	0.32	8.3	**.00**	0.15	3.2	.20
*ins‐30*	0.66	1.9	.10	0.13	2.1	.43	0.21	2.0	**.01**
*ins‐7*	0.71	4.2	**.00**	0.10	1.8	.40	0.18	3.0	**.00**
*N2*	0.62	3.4	–	0.12	1.5	–	0.14	2.1	–
*pdk‐1*	0.57	6.4	.07	0.34	5.7	**.00**	0.14	2.8	.22
*sgk‐1*	0.88	2.5	**.00**	0.05	0.9	.08	0.07	0.9	.23

Bolded values indicate p‐values that were less than 0.05.

### 2.9 A stochastic component contributes to movement decline

Consistent with the observations of Herndon et al. (2002) and Churgin et al. (2017), we observed variation in the onset and progression of movement decline within all strains (Figure 4). Worms that were long‐lived within a strain remained in Class A longer and entered Class B or Class C later (Figure 4). Notably, this was also observed in the short‐lived *sgk‐1* mutant which had extended Class A. We believe this surprising result may be explained by the observation from Mizunuma et al., 2014; that the *sgk‐1* lifespan is dependent on environmental context. Upon leaving Class A, an individual worm went on to Class B and subsequently Class C. Variation in this progression was observed (Figure 4). Further, the amount of time in Class B or C varied across individuals within a sample (Figure 4). These observations of within‐strain variation in movement are consistent with the observations of Churgin et al. (2017) where long‐lived or short‐lived animals share patterns of functional decline, regardless of genotype.

Contrary to Herndon et al. (2002), we observed reversal of class order (reversion of a later‐life movement phenotype to an earlier phenotype). While not common, we observed this in all mutants and in the control (Figure 4).

## 3 DISCUSSION

### 3.1 Long‐lived mutants do not have extended frailty

Research on aging biology has successfully identified various interventions that extend lifespan. A motivator of identifying such interventions is translation of those findings to humans. Model systems have been essential for this process; the question of whether health is also extended is being actively explored. The results seem to vary. Given the importance of the topic, further experimental attention is deserved. To address the question, “Do genetic manipulations that extend lifespan also extend healthspan?”, we monitored survival and movement longitudinally in nine of the insulin/insulin‐like signaling (IIS) pathway mutants in *C. elegans*. The mutants studied here include: *daf‐2(e1370), age‐1(hx546), pdk‐1(sa680), akt‐1(mg306), akt‐2(ok393), sgk‐1(ok538), ins‐7(ok1573), ins‐30(ok2343),* and *daf‐16(mu86)*. We used the three movement behavior classes championed by Herndon et al. (2002): Class A is a marker of biological health, Class B marks the onset of functional decline, and Class C indicates complete loss of homeostasis and a biologically frail state. We studied movement both in mass culture and in individual worms. This made it possible to assess movement and lifespan variation both across and within populations.

Our evaluations of movement differ from other studies in several ways: (i) we assessed nine IIS pathway mutants, (ii) the operative definition of frailty was more stringent than a 50% decline in function (a definition used by Bansal et al. (2015)), (iii) movement was measured longitudinally until 100% of the population died, (iv) both mass culture and individual worms were studied, and (v) event‐history diagrams were used, providing detailed visualization of longitudinal life‐history data (Carey et al., 1998).

An important finding of our study is that long‐lived mutant populations do not have extended frailty (Class C) compared to wild‐type. Moreover, populations of long‐lived mutants *daf‐2, age‐1, akt‐1,* and *pdk‐1* had reduced odds of frailty compared to N2. Significantly extended frailty throughout life was only observed in one mutant population, the short‐lived *ins‐30* mutant (Figure 3). The short‐lived mutant *daf‐16* trended toward extended frailty, but this did not reach statistical significance. The absence of an extended frailty period is contradictory to the medical model of healthspan, or the notion that frailty, or decrepitude, is extended as a result of extended longevity. In fact, population frailty was unaltered by genetic changes in lifespan.

We assessed movement decline longitudinally in individual worms. We calculated the number of days an individual worm spent in a given movement class. If frailty was extended in long‐lived mutants, we would expect to see an increase in the average number of days spent as Class C. We did not observe this for most long‐lived mutants. There was no difference in the number of days spent as Class C for *daf‐2, age‐1, akt‐2,* and *pdk‐1*. However, the average number of days spent as Class C was higher across *akt‐1* mutant individuals (Table 2). The different observation for *the akt‐1* mutant in the individual analysis compared to the population analysis may be controlling for lifespan. We did not control for lifespan in the individual analysis; if a mutant has a longer lifespan, there is an expected increase in the number of days in all movement classes. *ins‐7* and *ins‐30* mutants displayed an increase in the number of days as Class C, and both were short‐lived in our hands.

We then asked whether the proportion of life spent being frail was extended in IIS pathway mutants. From assessments of individual worm populations, we determined the proportion of life spent in a movement class. The average number of days spent in a class was normalized to the mean lifespan of the strain. Previous studies have normalized to maximal lifespan, which is known to be variable; our work further supports that notion. N2 worms spend an average of 14% of their life being frail. Long‐lived mutants *daf‐2, age‐1, akt‐2,* and *pdk‐1* did not increase the proportion of frailty (Class C), whereas the long‐lived *akt‐1* mutants spend an average of 21% of life being frail (Table 2, Figure 5). This increase was significant, although we did not control for longer lifespans in the individual analysis. Collectively, these observations of movement decline at both the population and individual levels in *C. elegans* mutants do not support the notion that an extension in life results in an extension of frailty.

### 3.2 Long‐lived mutants have extended mid‐life movement

We observed other characteristics of movement decline. Spontaneous (Class A) movement declined with age regardless of genotype. Most long‐lived mutant populations did not display differences in Class A movement compared to N2. For the *akt‐2* and *pdk‐1* mutants, there were significant interactions between genotype and age in explaining the variation in Class A movement. The rate of Class A decline for *akt‐2* and *pdk‐1* mutants differed from N2. *akt‐2* maintained Class A longer than N2, while *pdk‐1* lost Class A more quickly. In the individual analysis, *age‐1* and *akt‐1* have a larger proportion of life with Class A movement but other long‐lived mutants were similar to wild‐type (Figure 5). Again, a smaller sample size and not controlling for lifespan may explain the discrepancies between the population and individual analyses.

Short‐lived mutant populations *daf‐16, ins‐30,* and *ins‐7* lost Class A more rapidly than N2 (Figure 3). The *sgk‐1* mutant which was short‐lived in our hands but has been reported to be long‐lived (Hertweck et al., 2004) maintained Class A movement longer than N2. Interestingly, at the individual level, the short‐lived mutants did not have a smaller proportion of life as Class A. *ins‐7* and *sgk‐1* mutants had an extended proportion of life with Class A. Overall, an extension of “youthful” movement (Class A) was not observed to be a characteristic of movement decline for long‐lived mutant populations, but was observed for a smaller sample of *age‐1* and *akt‐1* mutants. Short‐lived mutant populations lose Class A more rapidly than wild‐type, but for *ins‐7* and *sgk‐1*, display a larger relative proportion of their life as Class A.

Provoked movement (Class B) represents a state of reduced biological function that occurs mid‐life and precedes frailty. At the population level, long‐lived mutants *daf‐2, age‐1, pdk‐1,* and *akt‐1* displayed greatly extended periods of Class B movement. For *daf‐2, age‐1,* and *pdk‐1*, the fractions of life in Class B were 400–600% that of N2. The *akt‐2* mutant was an exception and had reduced Class B movement at the population level. The rate of decline for Class B differed for *age‐1, akt‐1, daf‐2,* and *pdk‐1* and N2. For the short‐lived mutants *ins‐30, ins‐7,* and *sgk‐1,* the rate of decline of Class B also differed from N2. As expected from our population assessments, the proportion of life with Class B was extended in *daf‐2* and *pdk‐1* mutants and *akt‐2* mutants had a smaller proportion of life in Class B. *age‐1* did not show a statistically significant difference in Class B at the individual level, and this may be due to the small sample of individual *age‐1* worms. Of the short‐lived mutants examined here, *daf‐16* was the only mutant to spend a smaller proportion of life as Class B. Our study of mutations in the IIS pathway demonstrates that extending lifespan does not preserve youthful movement, but rather arrests the decline of mid‐life movement/preserves mid‐life movement.

This phenomenon of an extended mid‐life behavior in long‐lived mutants is consistent with previous reports of lethargy in the *daf‐2(e1370)* mutant which was attributed to a mild‐dauer phenotype (Gems et al., 1998). Bansal et al. (2015) and Hahm et al. (2015) did not include intermediate movement phenotypes, but other recent studies observed that an extended intermediate movement behavior was characteristic of both long‐lived mutants as well as long‐lived worms in wild‐type background (Churgin et al., 2017; Podshivalova et al., 2017; Zhang et al., 2016). Zhang et al. (2016) referred to this phenotype as “extended twilight” and Churgin et al. (2017) called the phenomena “decline then plateau” wherein long‐lived animals undergo rapid functional decline, then plateau.

These observations bring to the forefront an important point for discussion. While the operative definitions/classifications of movement phenotypes may vary, it is a consistent observation that the *daf‐2(e1370)* mutant and now other long‐lived mutants display an extended period of diminished movement. In contrast, short‐lived animals experience a rapid decline until death. Podshivalova et al. (2017) show that an extended period of reduced movement in *daf‐2* mutants is due to enhanced resistance to bacterial colonization that leads to premature death in wild‐type animals. The observations for other long‐lived mutants may be explained by a similar survivor benefit, a trade‐off between fitness and longevity, or inappropriate activation of the dauer pathway (Arantes‐Oliveira, Berman, & Kenyon, 2003).

### 3.3 Individual worms exhibit stochastic variation in movement decline and frailty

Variation in the rate of aging across individual worms has been observed in *C. elegans* (Bolanowski et al., 1981; Herndon et al., 2002; Johnson, 1987). Herndon et al. (2002) observed that loss of Class A movement occurred at different ages, even for individuals from an isogenic, age‐synchronized population that had shared a common environment from birth. Heterogeneity in movement decline in such a population demonstrates that senescence is due to random damage or failures (Herndon et al., 2002). These are stochastic components of the aging process, components other than genetics and environment that contribute to aging. Churgin et al. (2017) also observed within‐strain variation in movement decline in worms of wild‐type background. Their group demonstrated that long‐lived wild‐type worms share a pattern of movement decline with long‐lived mutants (Churgin et al., 2017). This observation was replicated for short‐lived animals. Churgin et al. (2017) concluded that stochastic factors that affect lifespan also affect healthspan.

In this study, the age of onset and progression of movement decline varied among individual worms within a population (Figure 4). There were general trends of movement decline; long‐lived individuals within a strain tended to have more days in Class A. When an individual left Class A, it went to Class B (except for rare instances where it went directly to Class C). Class C was observed shortly before death, supporting Class C as a marker of frailty. The event‐history plots (Figure 4) depict the variation in individual movement decline. Our observations of variation in movement decline in isogenic individual worms are consistent with Herndon et al. (2002) and Churgin et al. (2017) and are evidence for the stochasticity (random damage and failure) of nematode senescence.

### 3.4 Length of Class A is not consistently predictive of lifespan or later‐life movement

Using biomarkers of health has been useful for gaining insight into the relationship between age‐related declines that may be informative about the success of a longevity intervention. Retention of Class A movement has been used as a marker of extended healthspan (Herndon et al., 2002; Huang et al., 2004). We observed that long‐lived mutants generally retain Class A behavior longer (Figures 3 and 4). However, not all long‐lived mutants showed this and some short‐lived mutants (*sgk‐1* and *daf‐16)* did not show early loss of Class A. These data show that Class A does not predict lifespan. Further, the time in Class A was not predictive of time in Class B or C. Consistent with this, Podshivalova et al. (2017) report early‐life vigor for *daf‐2* mutants, including movement behavior, that was not correlated with the duration of later‐life decrepitude. In summary, the data show that early‐life movement (Class A) is not a good indicator of later‐life movement (Class B or C), thereby reducing the quality of its usage as a healthspan biomarker. We suggest that early‐life movement should not be used to define healthspan.

The variation within strains shows that age, genotype, and environment do not completely explain movement decay. Other groups observed the same variation in movement decline across individuals within a strain and concluded that stochastic events contribute to locomotion decline (Churgin et al., 2017; Herndon et al., 2002). Our results support this notion. However, we acknowledge that strains obtained from the CGC were not backcrossed to the Johnson Lab N2 prior to experiments; therefore, background effects cannot be ruled out. Additionally, researcher variability cannot be ruled out and may have contributed to the variation that we observed.

### 3.5 Class reversal

A characteristic of nematode movement decline observed in this study was class reversal (Class B to A, or C to B). Our data are not completely consistent with the report of Herndon et al. (2002), who failed to see class reversal. Though infrequent, class reversals were seen in all IIS mutants and wild‐type, suggesting that Class reversal is not strain specific and is a feature of nematode senescence. Class reversals indicate that some individuals are able to recover movement, if only briefly. However, differences between studies may explain why Herndon et al. (2002) reported a lack of class reversal. In our work, all worms were assigned a score at every assessment. Herndon et al. (2002) waited until the next scoring time to categorize worms that were difficult to score.

### 3.6 The paradox of *sgk‐1*

In this study, the *sgk‐1(ok538)* mutant was short‐lived. RNAi for the SGK‐1 protein has been reported to extend lifespan (Hertweck et al., 2004), but other studies report shortened lifespan for *sgk‐1* mutants (Chen et al., 2013; Xiao et al., 2013). The SGK‐1 protein complexes with AKT‐1/AKT‐2 to phosphorylate DAF‐16/FOXO*;* therefore, disruption of this process would be expected to extend lifespan. A search of the literature revealed that *sgk‐1* mutants can have shortened or lengthened lifespans depending on the combination of food and temperature culture conditions (Mizunuma et al., 2014). They show that SGK‐1 is activated in two pathways that affect lifespan oppositely. The mechanistic target of rapamycin complex 2 (mTORC2) and Rictor (RICT‐1) modulate longevity by causing SGK‐1 to inhibit the stress‐response transcription factor SKN‐1/Nrf in the intestine, shortening lifespan. In addition, RICT‐1/mTORC2 functions in neurons in an SGK‐1‐mediated pathway that increases lifespan at lower temperatures. RICT‐1/mTORC2 and SGK‐1 therefore oppose or accelerate aging depending upon the context in which they are active (Mizunuma et al., 2014). In our experiments, *sgk‐1* was short‐lived, had more Class A movement, and reduced frailty compared to N2. Perhaps the contextual involvement of *sgk‐1* in the IIS pathway could explain the paradox of a short‐lived but healthier mutant.

### 3.7 Healthspan, a nifty term for a complex phenomenon

Study of the age‐related loss of movement has been well documented in *C. elegans* and has been used as a marker of healthspan (Bolanowski et al., 1981; Duhon & Johnson, 1995; Glenn et al., 2004; Herndon et al., 2002; Honda & Honda, 2002; Hosono et al., 1980; Hsu, Feng, Hsieh, & Xu, 2009; Huang et al., 2004; Johnson, 1987; Shook, Brooks, & Johnson, 1996). Mechanisms of movement decline include structural and molecular changes resembling human sarcopenia (decrease in size and number of muscle cells, fraying of fibers, and loss of sarcomere integrity, Herndon et al., 2002), reduced neuronal signaling (Liu et al., 2013), and changes in exploratory behaviors (Hahm et al., 2015). The long‐lived *age‐1(hx546)* and two alleles of *daf‐2* delay age‐related changes to muscle nuclei (Herndon et al., 2002) and neuronal signaling (Liu et al., 2013) thereby promoting healthspan of the body muscle at the molecular level.

More recent investigation of age‐associated movement decline has been performed by several groups. Bansal et al. (2015) measured movement decline of long‐lived mutants (*daf‐2, eat‐2, ife‐2, and clk‐1)* by the distance travelled on solid media and the number of body bends per minute in liquid media. *daf‐2(e1370)* travelled farther distances than wild‐type from day 10 to day 20 (Bansal et al., 2015), but otherwise, no other differences between the mutants and wild‐type were observed. In their analysis of the movement data, Bansal et al. (2015) defined health as maintaining more than 50% of wild‐type function and frailty as having less than 50% wild‐type function. They calculated the chronological time (absolute number of days) and the “percent of the physiological lifespan” the worm spends in a healthy and frail state (the healthspan:gerospan ratio). Bansal et al. (2015) conclude that because the rate of mortality is reduced in long‐lived mutants while the rate of decline in the health parameters is not, long‐lived mutants do not exhibit a proportional increase in healthspan. Rather, they have an expansion of the frailty period (gerospan), when the data are normalized to maximal lifespan.

Following up on these observations, Hahm et al. (2015) assessed maximum movement velocity. Maximum velocity declined with age, correlated well with longevity, accurately reported movement ability, and, if measured in mid‐adulthood, was predictive of maximal lifespan. Movement velocity of *daf‐2(e1370)* mutants was proportional to their lifespan. Additionally, Hahm et al., 2015 reanalyzed the data from Bansal et al. (2015) employing human disease‐burden models. The human disease‐burden analysis revealed that the healthspan of *daf‐2(e1370)* mutants correlated with longevity extension, concluding that the *daf‐2(e1370)* mutant did not extend frailty.

Podshivalova et al. (2017) utilized both metrics of movement velocity and distance travelled in two *daf‐2* alleles. They show that *daf‐2(e1370)* and *daf‐2(e1368)* maintained movement longer than wild‐type, although the *daf‐2(e1370)* mutant had slower velocity and dauer‐like movement behaviors. Both *daf‐2* mutants were reported to have disproportionally extended late‐life decrepitude, supporting Bansal et al. (2015) that healthspan and lifespan can be genetically uncoupled. However, a beneficial trait leads to the decrepit phenotype. The *daf‐2* mutants have enhanced resistance to bacterial colonization that leads to premature death in wild‐type thereby preventing the manifestation of a decrepit phenotype in wild‐type animals (Podshivalova et al., 2017).

## 4 CONCLUSIONS

Although widely studied, methods of assessing body movement have varied qualitatively and quantitatively across groups. The qualitative and quantitative measures are highly correlated and often capture the same phenomena. However, there are limitations. Healthspan is a nifty term for a complex phenomenon and can be swayed by the experimenter's definition, methodology, focus, and intent. Based on this, two conceptual models of healthspan are emerging. The first one we call “the rate of aging model” which expects a positive relationship between healthspan and lifespan. The other we refer to as the “medical model” wherein life extension is not positively associated with health and results in frailty extension. This is an issue with which the field is currently grappling, with no clear solution. We argue that across healthspan studies, similar conclusions can be drawn. Some, but not all, long‐lived mutants perform better than wild‐type for body movement (and other healthspan parameters). When normalized to lifespan (maximal or mean), there is no clear and consistent elongation of healthspan or gerospan in long‐lived mutants. The conclusions of our studies are as follows:

1. Many long‐lived mutants have striking extension of a mid‐life movement behavior (Class B).
2. Lifespan‐extending mutations of the IIS pathway do not extend frailty. In fact, mutations that modulate lifespan do not greatly alter movement healthspan or frailty.
3. Variation in movement within isogenic, age‐matched populations is evidence that stochastic components affect nematode senescence, both in terms of lifespan and healthspan.
4. Our observations support the notion that healthspan parameters should be evaluated at older ages.
5. The *sgk‐1* mutant is short‐lived but extends youthful, early‐life movement.
6. Two models of healthspan are emerging contributing to conflicting results.

Overall, we find that the IIS class of longevity mutants extends health and lifespan and supports the conclusion of Hahm et al. (2015) that:

“The mechanistic study of coordinated health and life extension might allow the development of therapeutics to remedy end‐of‐life problems or to compress morbidity, decreasing health costs. *C. elegans IIS longevity mutants remain valuable tools in understanding the mechanisms by which we might achieve these goals.”*

## 5 METHODS

### 5.1 Strains

All strains were maintained using standard nematode conditions (incubated at 20 °C plated on 10 ml NGM plates spread with *E. coli*; OP50 (Brenner, 1974)). Strains were either ordered from the CGC or thawed from frozen laboratory stocks. We tested nine IIS mutants. All mutants had single‐gene mutations in an IIS pathway gene: *daf‐*2/IGF‐1 receptor, *age‐1/*PI3K orthologue, *pdk‐1/*AktVPKB kinase PDK‐1 orthologue, *akt‐1/*2/AKT1/2 orthologues*, sgk‐1/*SGK1 orthologue, *daf‐16/*FOXO transcription factor, *ins‐7/*IGF‐1‐like peptide, *ins‐30/*insulin‐like peptide.

The strains carrying the mutations of interest were as follows: CB1370 carrying the canonical allele *daf‐2(e1370)*, TJ1052 *age‐1(hx546),* CF1038 *daf‐16(mu86),* JT9609 *pdk‐1(sa680),* VC345 *sgk‐1(ok538*), BQ1 *akt‐1(mg306),* VC204 *akt‐2(ok393*), RB1388 *ins‐7(ok1573)*, and RB1809 *ins‐30(ok2343)*. N2 was the wild‐type control. *daf‐2, age‐1, pdk‐1,* and *akt‐1* carried substitution mutations, *akt‐2, daf‐16, ins‐7,* and *ins‐30* carried deletion mutations, and *sgk‐1* carried an insertion/deletion.

We selected strains based on the following criteria: 1) involved in IIS pathway, 2) reported in the literature as being long‐lived, 3) available from CGC or frozen laboratory stocks. In the case of *sgk‐1* and the insulin‐like peptide genes, the strains that had been reported to be long‐lived were RNAi mutants and not available from the CGC. In those cases, the mutant allele available from the CGC was used. Strains were not backcrossed to the Johnson Lab N2 strain prior to experiments.

### 5.2 Survival

Strain populations were age‐synchronized by hypochlorite prep (Stiernagle, 2006), and eggs were plated on OP50‐spotted 10 mm NGM plates. Animals were transferred to fresh plates every day for the first five days of life for monitoring of excessive larval deaths and separation of adults from progeny. No larval deaths were observed. After the first five days of life, populations were transferred to fresh plates every other day. Strains were plated at 10 worms/plate. Survival was scored every 1–3 days. Any animal that had a nonage‐related death (e.g., erupted vulva, killed, bagging, or lost) were censored.

### 5.3 Longitudinal assessment of movement in mutant populations

We measured movement behavior by scoring age‐synchronized, isogenic nematode strains for Class A, Class B, and Class C movement as classified by Herndon et al. (2002). Animals that are highly and spontaneously mobile are designated as Class A. Animals that are mobile but require a prodding stimulus from a metal wire are designated as Class B. Class C animals are only capable of moving their head or tail, or twitch in response to prodding. Class C animals do not move forward or backward. Animals that did not exhibit Class C movement and showed other signs of expiring were classified as dead. All animals begin with Class A movement. We scored animals every 1–3 days starting from the first day of adulthood until 100% of the population died.

### 5.4 Longitudinal assessment of movement in individual worms

Using the same movement scoring metric as listed above, we tracked movement across lifespan for individual worms. On a day when a worm transitioned out of Class A movement, it was placed onto its own portion of an agar plate so that it could be uniquely identified for subsequent scoring. Proportion of life spent in a movement class was determined by taking the maximal lifespan observed for a strain for a given experiment and dividing by the mean number of days spent in a movement Class.

### 5.5 Statistical analysis

Each mutant worm strain was evaluated in at least two experiments. Data from the multiple experiments were pooled for the analysis.

### 5.6 Survival

Survival differences between a mutant population and wild‐type were assessed using the log‐rank test. Log‐rank tests were run in R (R Core Team version 0.99.893) using the *survival* package (2.40–1). Median lifespan was determined using the quantile(surv) function in R that returns the quantiles of the survival curves (Therneau, 2.40‐1). Mean lifespan was determined as the day of 50% survival.

In individual worm populations, mean lifespan was determined by averaging the lifespans of individual worms.

### 5.7 Movement in mutant populations

To determine whether age and genotype affect movement class across strains, we used a binomial logistic regression. A logistic regression is a regression where the dependent variable is categorical. The binomial logistic regression model was used to fit the data using the statistical software R (0.99.893) and the *stats* package (version 2.12.0). The generalized linear model (GLM) was run with the binomial family and logit link function. This analysis included the predictor variables: age, genotype, and the interaction between age and genotype. Interactions between age and genotype were included in the model. Age was included as a predictor variable to account for strain differences in mean and maximal lifespan so that statistical differences in movement class were due to genotype effect. In addition, we tested for significant variability across replicate experiments and experimenters. The output of this model is the odds ratio for each mutant and movement class. For our movement studies, this ratio refers to the likelihood that the mutant will be in a given movement class at a given time compared with wild‐type. This model incorporates the designated covariates (age and genotype) and predicts how the odds ratio changes in response to the covariates. Movement decline is nonlinear; therefore, we used a binomial logistic model that included age and its interaction with genotype.

### 5.8 Movement in individual worms

A two‐tailed t‐test was used to determine whether the mean number of days spent in a movement class was significantly different between mutant and wild‐type. The two‐tailed t‐test was run in Microsoft Office Excel 2007 (12.0.6759.5000).

Event‐life‐history plots were generated following the methods outlined by Carey et al., 1998.

### 5.9 Figures

All figures were generated in R or Microsoft Office Excel 2007 (12.0.6759.5000).

## AUTHOR CONTRIBUTIONS

BLNS, DK, JC, and TEJ contributed to study design. BLNS collected the data and conducted the analyses. KK contributed to the analysis. PMT was critical for maintaining and/or generating nematode strains. BLNS wrote the initial version of the manuscript, and all authors critically commented on the initial versions of the manuscript and approved the final version of the manuscript for submission.

## CONFLICT OF INTEREST

None declared.
