# Supplementary material for: Movement decline across lifespan of Caenorhabditis elegans mutants in the insulin/insulin‐like signaling pathway
Source: Aging Cell. 2017 Dec 7;17(1):e12704. doi: 10.1111/acel.12704 (PMC5770877; doi:10.1111/acel.12704)
Supplement: Supplementary file 1 [file ACEL-17-na-s001.docx]

**Table S1. Effect of genotype on movement Class as a function of age.** Movement as a function of age was analyzed by binomial logistic regression. Every 1-3 days of adulthood the number of worms in a given movement Class (A, B, or C) were counted. The proportion of worms in a movement class was modeled by genotype, day of adulthood (age), and the interaction between genotype and day of adulthood (genotype:day). The binomial regression coefficients are listed as the estimates. The coefficients reflect the log change in the proportion of worms in a movement class with each observation period. Standard error represents the error of the regression coefficients. A strain’s odds of being in a given movement class relative to N2 are represented by the odds-ratio (OR). An OR greater than 1, indicates higher odds of a strain being in that movement class relative to N2. The 95% confidence interval of the regression coefficients are listed. Significance is indicated by the p-value.

| **Class A** | | | | | |
| --- | --- | --- | --- | --- | --- |
| **Coefficients** | **Estimate** | **Std. Error** | **p-value** | **OR** | **95% CI** |
| (Intercept) | 0.412140 | 0.057971 | 1.2e-12 | 1.00 | 1.35-1.70 |
| *age1* | -0.077807 | 0.110018 | 0.47943 | 1.62 | 0.75-1.15 |
| *akt1* | -0.005687 | 0.109627 | 0.95862 | 2.61 | 0.80-1.23 |
| *akt2* | -0.146547 | 0.125148 | 0.24160 | 1.27 | 0.68-1.10 |
| *daf16* | 0.017265 | 0.099450 | 0.86218 | 2.37 | 0.84-1.24 |
| *daf2* | 0.013437 | 0.084980 | 0.87436 | 2.40 | 0.86-1.20 |
| *ins30* | -0.007397 | 0.149686 | 0.96059 | 2.61 | 0.74-1.33 |
| *ins7* | 0.058681 | 0.141737 | 0.67886 | 1.97 | 0.80-1.40 |
| *pdk1* | -0.038918 | 0.111696 | 0.72752 | 2.07 | 0.77-1.20 |
| *sgk1* | -0.272624 | 0.103457 | 0.00841 | 1.01 | 0.62-0.93 |
| day | -0.106791 | 0.006983 | 2e-16 | 1.00 | 0.89-0.91 |
| *age1:*day | 0.012423 | 0.010767 | 0.24859 | 1.28 | 0.99-1.03 |
| *akt1:*day | -0.004254 | 0.012134 | 0.72588 | 2.07 | 0.97-1.02 |
| *akt2:*day | 0.051540 | 0.012516 | 3.8e-05 | 1.00 | 1.03-1.08 |
| *daf16*:day | -0.034644 | 0.013725 | 0.0116 | 1.01 | 0.94-0.99 |
| *daf2:*day | -0.009165 | 0.008716 | 0.2930 | 1.34 | 0.97-1.01 |
| *ins30:*day | -0.142728 | 0.025025 | 1.2e-08 | 1.00 | 0.86-0.91 |
| *ins7:*day | -0.038996 | 0.017903 | 0.02939 | 1.02 | 0.93-1.00 |
| *pdk1:*day | -0.060573 | 0.013632 | 8.9e-06 | 1.00 | 0.92-0.97 |
| *sgk1:*day | 0.072254 | 0.011747 | 7.7e-10 | 1.00 | 1.05-1.10 |

| **Class B** | | | | | |
| --- | --- | --- | --- | --- | --- |
| **Coefficients** | **Estimate** | **Std. Error** | **p-value** | **OR** | **95% CI** |
| (Intercept) | -3.08389 | 0.13410 | 2.0e-16 | 1.00 | 0.04-0.06 |
| *age1* | 1.27616 | 0.19266 | 3.5e-11 | 1.00 | 2.46-5.23 |
| *akt1* | 0.36000 | 0.23959 | 0.13294 | 1.14 | 0.90-2.29 |
| *akt2* | -1.35004 | 0.43890 | 0.00210 | 1.00 | 0.11-0.61 |
| *daf16* | 0.13087 | 0.22234 | 0.55612 | 1.74 | 0.74-1.76 |
| *daf2* | 1.72358 | 0.15264 | 2.0e-16 | 1.00 | 4.16-7.56 |
| *ins30* | 1.89388 | 0.19251 | 2.0e-16 | 1.00 | 4.56-9.69 |
| *ins7* | 0.49347 | 0.26549 | 0.06307 | 1.07 | 0.97-2.76 |
| *pdk1* | 1.86726 | 0.17268 | 2.0e-16 | 1.00 | 4.61-9.08 |
| *sgk1* | -1.78431 | 0.39753 | 7.2e-06 | 1.00 | 0.08-0.37 |
| day | 0.10111 | 0.01046 | 2.0e-16 | 1.00 | 1.08-1.13 |
| *age1:*day | -0.06550 | 0.01319 | 6.9e-07 | 1.00 | 0.91-0.96 |
| *akt1:*day | -0.03215 | 0.01664 | 0.05328 | 1.05 | 0.94-1.00 |
| *akt2:*day | 0.04231 | 0.02653 | 0.11073 | 1.12 | 0.99-1.10 |
| *daf16*:day | 0.01208 | 0.02016 | 0.54896 | 1.73 | 0.97-1.05 |
| *daf2:*day | -0.07279 | 0.01093 | 2.8e-11 | 1.00 | 0.91-0.95 |
| *ins30:*day | -0.05265 | 0.01769 | 0.00291 | 1.03 | 0.92-0.98 |
| *ins7:*day | 0.03182 | 0.02142 | 0.13747 | 1.15 | 1.00-1.08 |
| *pdk1:*day | -0.07133 | 0.01278 | 2.4e-08 | 1.00 | 0.91-0.95 |
| *sgk1:*day | 0.09397 | 0.02996 | 0.00171 | 1.00 | 1.04-1.16 |

| **Class C** | | | | | |
| --- | --- | --- | --- | --- | --- |
| **Coefficients** | **Estimate** | **Std. Error** | **p-value** | **OR** | **95% CI** |
| (Intercept) | -4.63626 | 0.17485 | 2.0e-16 | 1.00 | 0.01-0.01 |
| *age1* | 0.11278 | 0.32570 | 0.72913 | 2.07 | 0.59-2.12 |
| *akt1* | 0.56918 | 0.30634 | 0.06316 | 1.07 | 0.97-3.22 |
| *akt2* | -0.50079 | 0.47528 | 0.29203 | 1.34 | 0.24-1.54 |
| *daf16* | 0.43174 | 0.27456 | 0.11584 | 1.12 | 0.90-2.64 |
| *daf2* | -0.70695 | 0.27720 | 0.01076 | 1.01 | 0.29-0.85 |
| *ins30* | 0.74551 | 0.33636 | 0.02667 | 1.03 | 1.09-4.07 |
| *ins7* | -0.78612 | 0.51688 | 0.12829 | 1.14 | 0.17-1.25 |
| *pdk1* | -0.18726 | 0.32387 | 0.56312 | 1.76 | 0.44-1.56 |
| *sgk1* | -1.37987 | 0.49592 | 0.00540 | 1.01 | 0.10-0.67 |
| day | 0.23005 | 0.01124 | 2.0e-16 | 1.00 | 1.23-1.29 |
| *age1:*day | -0.07663 | 0.01632 | 2.7e-06 | 1.00 | 0.90-0.96 |
| *akt1:*day | -0.05502 | 0.01767 | 0.00185 | 1.00 | 0.91-0.98 |
| *akt2:*day | -0.02283 | 0.02647 | 0.38829 | 1.47 | 0.93-1.03 |
| *daf16*:day | 0.03869 | 0.02100 | 0.06538 | 1.07 | 0.10-1.08 |
| *daf2:*day | -0.09741 | 0.01304 | 7.9e-14 | 1.00 | 0.88-0.93 |
| *ins30:*day | -0.01474 | 0.02577 | 0.56739 | 1.76 | 0.94-1.04 |
| *ins7:*day | 0.04053 | 0.03391 | 0.23210 | 1.26 | 0.97-1.11 |
| *pdk1:*day | -0.04624 | 0.01720 | 0.00718 | 1.01 | 0.92-0.99 |
| *sgk1:*day | 0.04905 | 0.03376 | 0.14624 | 1.16 | 0.98-1.12 |

**Table S2. Mean number of days in a movement class.** Every 1-3 days of adulthood individual worms were scored by movement class. Worms were kept separate so that changes in movement could be followed longitudinally. The mean number of days for each class was determined. The p-values represent results of a two-tailed t-test comparing mean number of days for each class between a mutant strain and the wild-type (N2).

| Strain | Class | Mean  (days) | St Dev  (days) | p-value | n |
| --- | --- | --- | --- | --- | --- |
| *N2* | A | 9.3 | 3.4 | - | 59 |
|  | B | 1.8 | 1.5 | - | 59 |
|  | C | 2.1 | 1.8 | - | 59 |
| *ins-7* | A | 15.1 | 4.3 | 0.00 | 16 |
|  | B | 2.2 | 1.8 | 0.39 | 16 |
|  | C | 3.9 | 3.1 | 0.00 | 16 |
| *ins-30* | A | 10.7 | 2.0 | 0.10 | 18 |
|  | B | 2.2 | 2.1 | 0.43 | 18 |
|  | C | 3.4 | 2.1 | 0.01 | 18 |
| *daf-2* | A | 10.2 | 6.2 | 0.38 | 25 |
|  | B | 6.0 | 8.3 | 0.00 | 25 |
|  | C | 2.8 | 3.2 | 0.20 | 25 |
| *age-1* | A | 14.4 | 2.1 | 0.00 | 9 |
|  | B | 1.4 | 1.9 | 0.50 | 9 |
|  | C | 1.8 | 2.2 | 0.68 | 9 |
| *pdk-1* | A | 11.3 | 6.4 | 0.07 | 21 |
|  | B | 6.8 | 5.7 | 0.00 | 21 |
|  | C | 2.7 | 2.8 | 0.22 | 21 |
| *akt-1* | A | 10.9 | 4.1 | 0.02 | 55 |
|  | B | 2.4 | 2.0 | 0.10 | 55 |
|  | C | 4.0 | 3.5 | 0.00 | 55 |
| *akt-2* | A | 10.8 | 5.5 | 0.07 | 32 |
|  | B | 1.1 | 1.2 | 0.00 | 32 |
|  | C | 1.5 | 1.8 | 0.12 | 32 |
| *sgk-1* | A | 16.6 | 2.5 | 0.00 | 11 |
|  | B | 1.0 | 0.9 | 0.08 | 11 |
|  | C | 1.4 | 0.9 | 0.23 | 11 |
| *daf-16* | A | 9.6 | 3.6 | 0.70 | 38 |
|  | B | 1.0 | 1.6 | 0.01 | 38 |
|  | C | 2.1 | 2.2 | 1.00 | 38 |
